# Serum Fatty Acid Composition Balance by Fuzzy C-Means Method in Individuals with or without Metabolic Dysfunction-Associated Fatty Liver Disease

**DOI:** 10.3390/nu15040809

**Published:** 2023-02-04

**Authors:** Yuka Nagase, Takao Satoh, Keiichi Shigetome, Naoto Tokumaru, Erika Matsumoto, Kazunori D. Yamada, Tadashi Imafuku, Hiroshi Watanabe, Toru Maruyama, Yasuhiro Ogata, Minoru Yoshida, Junji Saruwatari, Kentaro Oniki

**Affiliations:** 1 Division of Pharmacology and Therapeutics, Graduate School of Pharmaceutical Sciences, Kumamoto University, Kumamoto 862-0973, Japan; 2 Kumamoto Industrial Research Institute, Kumamoto 862-0901, Japan; 3 Unprecedented-Scale Data Analytics Center, Tohoku University, Sendai 980-8578, Japan; 4 Department of Molecular Pathophysiology, Institute of Advanced Medicine, Wakayama Medical University, Wakayama 641-8509, Japan; 5 Department of Biopharmaceutics, Graduate School of Pharmaceutical Sciences, Kumamoto University, Kumamoto 862-0973, Japan; 6 Japanese Red Cross Kumamoto Health Care Center, Kumamoto 861-8520, Japan

**Keywords:** fatty acid, fatty acid composition balance, fuzzy c-means method, clustering, metabolic dysfunction-associated fatty liver disease

## Abstract

Circulating fatty acid composition is assumed to play an important role in metabolic dysfunction-associated fatty liver disease (MAFLD) pathogenesis. This study aimed to investigate the association between the overall balance of serum fatty acid composition and MAFLD prevalence. This cross-sectional study involved 400 Japanese individuals recruited from a health-screening program. We measured fatty acids in serum lipids using gas chromatography–mass spectrometry. The serum fatty acid composition balance was evaluated using fuzzy c-means clustering, which assigns individual data points to multiple clusters and calculates the percentage of data points belonging to multiple clusters, and serum fatty acid mass%. The participants were classified into four characteristic subclasses (i.e., Clusters 1, 2, 3, and 4), and the specific serum fatty acid composition balance (i.e., Cluster 4) was associated with a higher MAFLD prevalence. We suggest that the fuzzy c-means method can be used to determine the circulating fatty acid composition balance and highlight the importance of focusing on this balance when examining the relationship between MAFLD and serum fatty acids.

## 1. Introduction

Metabolic dysfunction-associated fatty liver disease (MAFLD) is a newly proposed definition of fatty liver disease (FLD) [1,2] that is diagnosed in individuals with fatty liver who meet one of the three criteria: (1) overweight/obesity; (2) type 2 diabetes; and (3) at least two metabolic risk abnormalities among increased waist circumference (WC), elevated blood pressure, increased triglycerides, decreased high-density lipoprotein cholesterol (HDL-C), prediabetes, insulin resistance, and increased high-sensitivity C-reactive protein level [1,2]. MAFLD is a hepatic manifestation of multiple metabolic diseases influencing hepatic lipid accumulation, inflammation, and fibrosis, and its underlying causes, symptoms, course, and outcomes are heterogeneous [1,2]. Compared to the commonly used definition of nonalcoholic fatty liver disease (NAFLD), the new definition has several advantages [3,4,5,6]. For example, the MAFLD definition can be used to better identify hepatic steatosis in individuals at high risk of hepatic and extrahepatic complications [3,4,5,6]. In addition, it has been shown to capture more patients who may benefit from assessment of genetic risk factors for fatty liver [3,4,5,6].

Lipidomics research provides meaningful data on the lipid profiles involved in fatty liver pathology and their regulation as potential preventive and therapeutic targets; the popularity of this field has grown in recent years. Fatty acids are used in the liver for triglyceride synthesis, and they play an essential role in the pathogenesis of FLD [7]. Fatty acids are classified based on the length of carbon chains and nature of double bonds [8]. Saturated fatty acids (SFAs) have no double bonds in the acyl chain [8]. In contrast, unsaturated fatty acids are fatty acid species with one or more insertions and are classified as monounsaturated fatty acids (MUFAs) with one double bond and polyunsaturated fatty acids (PUFAs) with multiple double bonds [8]. The amount of each fatty acid species, i.e., the fatty acid composition, in serum and plasma has been suggested as a potential determinant of energy metabolism, insulin resistance pathology in the liver, and intracellular lipid accumulation [9]. SFAs are considered a major fatty acid species involved in lipotoxicity. Previous evidence suggests that intrahepatic accumulation of SFAs may cause cellular dysfunction and upregulation of pro-inflammatory pathways, eventually leading to hepatic triglyceride accumulation [8,10]. Triglycerides are primarily composed of MUFAs, which are considered less lipotoxic than SFAs [8,10]. PUFAs, mainly omega-3 PUFAs, are expected to be beneficial for preventing and treating FLD via mechanisms that induce fatty acid oxidation, suppress *de novo* lipogenesis, and inhibit transcriptional regulators [11]. Meanwhile, the amount of each fatty acid species can affect each other, e.g., palmitic acid (C16:0) is converted to palmitoleic acid (C16:1 omega-7) with an additional double bond by stearoyl-CoA desaturase (SCD) 1 or stearic acid (C18:0) with an increased fatty acid chain by elongation of the long-chain fatty acid family member (ELOVL) 6; linoleic acid (C18:2 omega-6) is converted to arachidonic acid (C20:4 omega-6) by fatty acid desaturase (FADS) 2, ELOVL5, and FADS1; and PUFAs decrease the conversion of SFAs to MUFAs by reducing SCD1 expression [12,13]. In addition, individual and environmental factors (e.g., age, sex, lifestyle, disease status, and genetic factors) influence the amount of each fatty acid species and the activities of fatty acid-converting enzymes [7,8,13]. Therefore, when evaluating the association between serum fatty acid composition and FLD, it may be necessary to consider the amount of each fatty acid and the fatty acid composition balance. Patients with and without NAFLD have different fatty acid compositions in serum or plasma, as observed in previous human studies [8,14,15]. However, the total fatty acid concentration in serum or plasma is higher in patients with NAFLD than in those without it [8,14,15]; thus, it is unclear whether the circulating fatty acid composition balance is related to FLD. Furthermore, to date, no method has been established for assessing circulating fatty acid composition balance.

Therefore, in this study, we investigated the association between serum fatty acid composition balance and MAFLD prevalence in the Japanese population. Clustering is a method for evaluating serum fatty acid composition balance; however, clustering (e.g., the k-means method) is insufficient because it does not provide detailed information on serum fatty acid composition balance at individual data points. The fuzzy c-means method is a clustering method that is used to assign individual data points to multiple clusters and calculate the percentage of data points belonging to each cluster [16,17]. Hence, the fuzzy c-method was used in this study to evaluate serum fatty acid composition balance. This method is expected to clarify the extent to which individual data points are distinguished by serum fatty acid composition balance across multiple clusters and to provide detailed information on cluster proximity.

## 2. Methods

### 2.1. Study Participants

This cross-sectional case-control study involved 400 participants who were recruited from the Japanese Red Cross Kumamoto Health Care Center. The Ethics Committee of the Faculty of Life Sciences, Kumamoto University, and the Japanese Red Cross Kumamoto Health Care Center approved the study protocol. All participants provided written informed consent before enrollment in the study.

### 2.2. Measurement of Serum Fatty Acids

Since serum fatty acid composition is greatly influenced by the most recent meal [18,19], blood samples of the participants were collected in the morning after an overnight fasting in a tube containing disodium ethylenediaminetetraacetate. First, fatty acids in the serum lipids, including glycerides, phospholipids, free fatty acids, and cholesterol esters, were methylated using a fatty acid methylation kit (Nacalai Tesque, Kyoto, Japan). Gas chromatography–mass spectrometry (GC-MS) (5975C inert XL MSD; Agilent) was used for measuring the concentrations of myristic acid (C14:0), palmitic acid (C16:0), stearic acid (C18:0), palmitoleic acid (C16:1 omega-7), oleic acid (C18:1 omega-9), linoleic acid (C18:2 omega-6), alpha- and gamma-linolenic acid (C18:3), arachidonic acid (C20:4 omega-6), eicosapentaenoic acid (C20:5 omega-3), and docosahexaenoic acid (C22:6 omega-3). The instrument was equipped with a capillary column (0.25 µm × 30 m × 0.25 mm, VF-WAXms; Agilent). Nonadecanoic acid (C19:0) (Sigma-Aldrich, St. Louis, MO, USA) was used as the internal standard for the following reasons: (1) it is chemically stable, (2) it elutes close to the fatty acids analyzed in this study, (3) it separates almost completely from all components in the sample, and (4) it is virtually absent in human blood [20].

### 2.3. Genotyping

Genomic DNA was extracted from whole blood samples using a DNA purification kit (FlexiGene DNA kit; QIAGEN, Hilden, Germany). The *patatin-like phospholipase domain-containing 3 gene* (*PNPLA3*) rs738409 C > G (encoding c.444C > G, I148M) polymorphism has been recognized as a major genetic risk factor for the development and progression of NAFLD [21,22]. Therefore, to adjust for the effects of the *PNPLA3* rs738409 polymorphism in multivariable analysis of the MAFLD prevalence, the polymorphism was genotyped by real-time TaqMan allelic discrimination assay (Applied Biosystems, Waltham, MA, USA) (Assay No. C_7241_10). For the genotyping, pooled DNA from healthy volunteers with known genotypes and a negative control (water) were included as internal controls to ensure genotyping quality.

### 2.4. Diagnosis of MAFLD

Hepatic ultrasonography scanning was used for diagnosing FLD based on four criteria: a diffuse hyperechoic echotexture, an increased echotexture compared to the kidneys, vascular blurring, and deep attenuation [23]. After a radiologist diagnosed FLD, a physician reviewed the images to assess the accuracy and reproducibility of the diagnosis. For the diagnosis of MAFLD, according to previous reports [1,2], one or more of the following conditions coexisted with FLD diagnosed by hepatic ultrasonography scanning: (1) body mass index (BMI) ≥ 23 kg/m^2^; (2) presence of type 2 diabetes; (3) BMI < 23 kg/m^2^ along with the presence of two of the following metabolic risk abnormalities: WC ≥ 90 cm in men and ≥ 80 cm in women; blood pressure ≥ 130/85 mmHg or use of antihypertensive medicines; triglycerides (TGs) ≥ 150 mg/dL or use of dyslipidemia medications; HDL-C ≥ 40 mg/dL in men and ≥ 50 mg/dL in women; fasting blood glucose (FBG) = 100–125 mmol/L or HbA1c = 5.7–6.4%; high-sensitivity C-reactive protein > 2.0 mg/L. The fibrosis (FIB)-4 index, an indicator of fibrosis in FLD subjects, was calculated from age, platelet count, aspartate aminotransferase (AST), and alanine aminotransferase (ALT) using the following formula: [age × AST (IU/L)] / [(platelet count (10^9^) × √ALT (IU/L)] [24].

### 2.5. Data Collection

The laboratory tests were performed using the standard methods of the Japan Society of Clinical Chemistry. Type 2 diabetes was diagnosed on the basis of the history of the patient and the criteria recommended by the American Diabetes Association Expert Committee. Information on dietary habits was collected by means of a questionnaire.

### 2.6. Statistical Analysis

The data are expressed as the mean ± standard deviation or median (range) for continuous variables and as proportion for categorical variables. Fisher’s exact test or the Fisher–Freeman–Halton test was used for comparing categorical variables. Student’s *t*-test or one-way ANOVA was used for comparing continuous parametric values, and the Mann–Whitney *U* test or Kruskal–Wallis test was used for comparing continuous nonparametric values. The Mantel–Haenszel test for trend or Jonckheere–Terpstra trend test was used for trend testing of categorical or continuous variables, respectively.

The participants were clustered on the basis of the standardized mass% of the 10 serum fatty acids to represent the serum fatty acid composition balance. For clustering, we used the fuzzy c-means method that assigns individual data points to multiple clusters and calculates the percentage of data points belonging to multiple clusters [16,17]. The number of clusters was determined to be four using the Elbow method [25]. The *m* value (i.e., the fuzzy weighting exponent) was set to 2.0 [16,17]. In the clustering method, data points of the participants were plotted on the basis of the standardized mass% of the 10 serum fatty acids, and the centroids of the four clusters were determined randomly at the beginning. Next, their centroids were recalculated from the Euclidean distances between the data points. Subsequently, all cluster percentages at each data point of the participants were calculated on the basis of the Euclidean distances from the centroids of the clusters. Furthermore, the above calculations (i.e., cluster centroids and cluster percentages belonging to the data points) were repeated and terminated when no changes were observed in the results. The study participants were classified into the clusters with the highest percentage of belonging among the four clusters. We used principal component analysis to reduce the dimensions of the serum fatty acid composition balance and constructed a two-dimensional graph using the first two principal components to visualize the proximity of the four clusters.

The association between MAFLD prevalence and the four clusters was analyzed by multivariable logistic regression analysis. This association was measured as odds ratios (ORs) and 95% confidence intervals (95% CIs) for the prevalence of MAFLD in Clusters 2, 3, and 4 compared to Cluster 1; ORs were adjusted for age, sex, BMI, total concentration of fatty acids, and *PNPLA3* rs738409 polymorphism using the forced entry method. When developing the multivariable logistic regression model for MAFLD, the probability (Pr) of prevalence of MAFLD was expressed as the following inverse logit function:

$$Pr=\frac{e^{logit\left(Pr\right)}}{1+e^{logit\left(Pr\right)}}$$

Logit (Pr) values describing the linear relationship between prevalence of MAFLD and the covariates were calculated using the following equation:

$$logit\left(Pr\right)=\beta_{0}+\beta_{1}X_{1}+\beta_{2}X_{2}+\beta_{3}X_{3}+\cdots +\beta_{n}X_{n}+\cdots$$

where β_n_ represents the standardized coefficients; x_n_ respresents the covariates.

The ORs of covariates were calculated using the following equation:

$$OR=e^{\beta_{n}}$$

A *p* < 0.05 value was considered statistically significant. Multiple comparisons were corrected using Bonferroni’s method, and *p* values < 0.05/n were considered statistically significant after correcting for the number of comparisons made. The fuzzy c-means method was performed using the scikit-fuzzy library (version 0.4.2, Python Software Foundation, Wilmington, NC, USA) of Python (version 3.9.12, Python Software Foundation, Wilmington, NC, USA). The SPSS software package (version 28.0; IBM Japan Inc., Tokyo, Japan) was used for all other statistical analyses.

## 3. Results

### 3.1. Participant Characteristics

Table 1 shows the clinical characteristics of participants with and without MAFLD. The participants with and without MAFLD had different values for age, BMI, WC, HbA1c, FBG, HDL-C, low-density lipoprotein cholesterol (LDL-C), TGs, AST, ALT, gamma-glutamyl transferase (GGT), and smoking status (Table 1). *PNPLA3* C/C, C/G, and G/G genotype frequencies of the participants were 27.3%, 55.0%, and 17.8%, respectively. The *PNPLA3* genotype frequencies were in the Hardy–Weinberg equilibrium (*p* > 0.05), and they differed between participants with and without MAFLD (Table 1). Similar to previous studies [21,22], bi-variable logistic regression analysis revealed that the frequency of MAFLD was higher in participants with the *PNPLA3* C/G and G/G genotypes than in those with the C/C genotype, with ORs (95% CI) of 2.04 (1.21–3.44) and 2.19 (1.14–4.20), respectively.

### 3.2. Clustering Based on a Standardized Concentration of 10 Serum Fatty Acids

**Figure 1:**
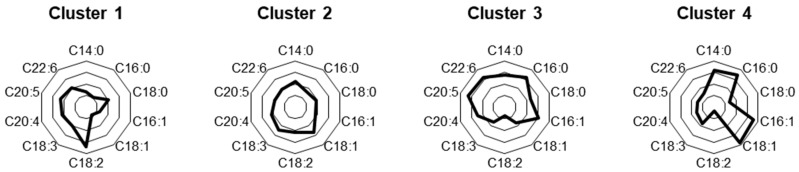
Radar charts of standardized mass% of the 10 serum fatty acids at the centroids of the four clusters.

**Figure 2:**
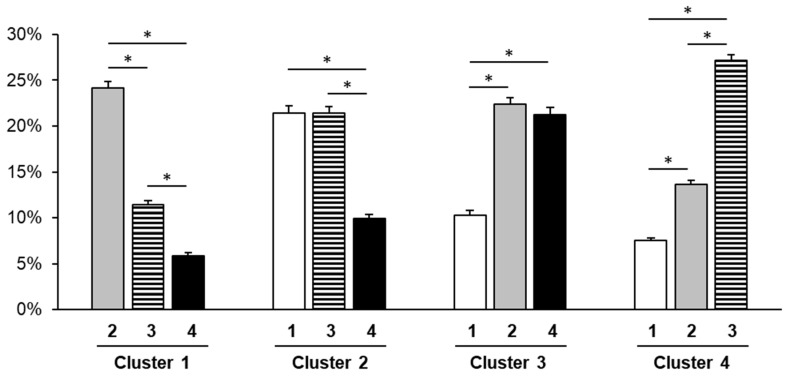
Percentages of belonging to the other clusters within each cluster. * *p* < 0.001.

**Figure 3:**
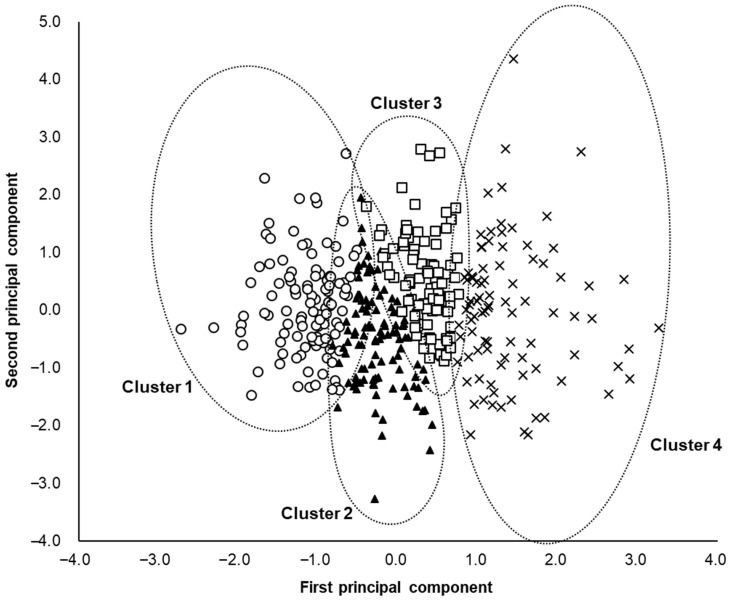
Principal component analysis. The x- and y-axes represent the first and second principal component, respectively. Data points belonging to clusters 1, 2, 3, and 4 are indicated by open circles, closed triangles, open squares, and crosses, respectively.

The mass% of each serum fatty acid in the participants was calculated by dividing the amount of each serum fatty acid (μg/mL) by the total serum fatty acid content (μg/mL). The fuzzy c-means method was used for dividing the 400 participants into four characteristic subclasses based on the standardized mass% of the 10 serum fatty acids. Table 2 shows the differences in the mass% of the 10 serum fatty acids and the total concentration of serum fatty acids among the four clusters. Figure 1 shows the radar charts of the mass% of the 10 serum fatty acids at the centroids of the four clusters. At the centroid of Cluster 1, high mass% of linoleic acid (C18:2 omega-6) and low mass% of myristic acid (C14:0), palmitic acid (C16:0), palmitoleic acid (C16:1 omega-7), and oleic acid (C18:1 omega-9) were observed (Figure 1). At the centroid of Cluster 2, the mass% of all fatty acids was generally average (Figure 1). At the centroid of Cluster 3, high mass% of palmitic acid (C16:0), palmitoleic acid (C16:1 omega-7), eicosapentaenoic acid (C20:5 omega-3), and docosahexaenoic acid (C22:6 omega-3), and low mass% of linoleic acid (C18:2 omega-6) were observed (Figure 1). At the centroid of Cluster 4, high mass% of myristic acid (C14:0), palmitic acid (C16:0), palmitoleic acid (C16:1 omega-7), oleic acid (C18:1 omega-9), and low mass% of stearic acid (C18:0), linoleic acid (C18:2 omega-6), eicosapentaenoic acid (C20:5 omega-3), and docosahexaenoic acid (C22:6 omega-3) were observed (Figure 1). To investigate the proximity of clusters, the percentages of other clusters within each cluster were compared (Figure 2). In Cluster 1, the percentages were higher in the order of Cluster 2 > Cluster 3 > Cluster 4 (Figure 2). In Cluster 2, the percentages of Clusters 1 and 3 were higher than that of Cluster 4 (Figure 2). In Cluster 3, the percentages of Clusters 2 and 4 were higher than that of Cluster 1 (Figure 2). In Cluster 4, the percentages were higher in the order of Cluster 3 > Cluster 2 > Cluster 1 (Figure 2). Figure 3 shows the fatty acid composition balance expressed by the principal component analysis in terms of the first two principal components. Table S1 shows the principal component scores of the fatty acids.

Table 3 shows the differences in the clinical characteristics among the four clusters. BMI, WC, HDL-C, TGs, AST, ALT, GGT, alcohol intake, and MAFLD frequency differed among the clusters (Table 3). Moreover, the trend test revealed that the clusters in decreasing order of MAFLD prevalence are as follows: Cluster 4 > Cluster 3 > Cluster 2 > Cluster 1 (Table 3). Table S2 shows the results comparing the information on dietary habits among the clusters. There were differences in the frequency of eating fruits and sweets among the clusters (Table S2). Table S3 shows the differences in liver function test values and FIB-4 index among the four clusters in subjects with MAFLD. GGT and FIB-4 index were different between the four clusters (Table S3). Moreover, trend test results showed that the clusters in decreasing order of AST and GGT levels were as follows: Cluster 4 > Cluster 3 > Cluster 2 > Cluster 1 (Table S3).

### 3.3. Multivariable Analysis of the Association between the MAFLD Prevalence and Clusters

The prevalence of MAFLD differed among the four clusters (Table 3). Therefore, we used multivariable logistic regression analysis to examine the association of the MAFLD prevalence with each cluster (Table 4). The prevalence of MAFLD was higher in Cluster 4 than in Cluster 1, independent of age, sex, BMI, total concentration of fatty acids, and *PNPLA3* rs738409 polymorphism (Table 4). In contrast, no difference was observed in the prevalence of MAFLD between Clusters 2 and 3 and Cluster 1 (Table 4).

## 4. Discussion

This study represented the findings based on the serum fatty acid composition balance by clustering using the fuzzy c-means method. The participants were classified into four clusters on the basis of the serum fatty acid composition balance, and the proximity of these clusters was investigated. Moreover, we showed that the specific serum fatty acid composition balance (i.e., Cluster 4) was associated with the MAFLD prevalence, independent of several confounding factors (i.e., age, sex, BMI, total serum fatty acid concentration, and *PNPLA3* rs738409 polymorphism). These findings suggest a useful method for explaining the circulating fatty acid composition balance and its importance in MAFLD.

The fuzzy c-means method was used in this study for classifying the serum fatty acid composition balance into four characteristic patterns. We examined the relationship between these patterns and MAFLD prevalence. The fuzzy c-means method incorporates fuzziness into clustering and calculates the percentages of belonging to multiple clusters at individual data points [16,17]. The fuzzy c-means method can be used to provide information on the proximity of individual data points to other clusters in addition to the cluster to which it belongs on the basis of the percentages of individual data points belonging to multiple clusters. Comparison of the percentages of belonging to the other clusters within each cluster suggests that the serum fatty acid composition balances were close between Clusters 1 and 2, Clusters 2 and 3, and Clusters 3 and 4 (Figure 2). Additionally, the principal component analysis findings suggest the proximity of Clusters 1 and 2, Clusters 2 and 3, and Clusters 3 and 4 (Figure 3). We speculate that the serum fatty acid composition balance frequently shifted between Clusters 1 and 2, Clusters 2 and 3, and Clusters 3 and 4; however, further longitudinal studies are needed to confirm this theory.

Information on the proximity to each cluster at individual data points obtained using the fuzzy c-means method may also be useful in characterizing the serum fatty acid composition balance of the participants individually. Further studies are needed to clarify whether lifestyle modification can change the serum fatty acid composition balance pattern and whether such changes are effective for MAFLD prevention, treatment, or both. However, the findings of fuzzy c-means clustering may be useful in the future for proposing individualized prevention strategies for MAFLD, focusing on the serum fatty acid composition balance.

Cluster 4 was strongly associated with MAFLD prevalence, regardless of age, sex, BMI, total serum fatty acid concentration, and *PNPLA3* rs738409 polymorphism (Table 4). This finding emphasizes the significance of evaluating serum fatty acid composition in terms of individual serum fatty acid concentration and balance. The serum fatty acid composition balance of Cluster 4 was characterized by a high mass% of SFAs (myristic acid (C14:0) and palmitic acid (C16:0)) and MUFAs (palmitoleic acid (C16:1 omega-7) and oleic acid (C18:1 omega-9)) and a low mass% of omega-3 PUFAs (eicosapentaenoic acid (C20:5 omega-3) and docosahexaenoic acid (C22:6 omega-3)), steric acid (C18:0), and linoleic acid (C18:2 omega-6) (Table 2 and Figure 1). Previous human studies have found that patients with NAFLD have higher levels of SFAs and MUFAs in serum or plasma than those who do not have NAFLD [8,14,15]. SFA-enriched diets have been reported to increase hepatic triglycerides [26,27], which has been linked to increased lipolysis in adipose tissue and fatty acid transfer to the liver [26]. In contrast, MUFA-enriched diets have been reported to reduce intrahepatic triglyceride levels and improve hepatic and total insulin sensitivity [28,29,30]. In addition to SFA-rich diets, lipolysis, *de novo* synthesis, or both influence SFA increase [7]. However, it has been reported to increase SCD1 activity to avoid SFA-induced hepatotoxicity and increase MUFAs [12]. Indeed, while the mass% of stearic acid (C18:0) was very low in Cluster 4, the mass% of oleic acid (C18:1 omega-9) was very high (Table 2 and Figure 1), suggesting that most of the stearic acid (C18:0) may have been converted to oleic acid (C18:1 omega-9) due to increased SCD1 activity. Moreover, a low mass% of eicosapentaenoic acid (C20:5 omega-3), docosahexaenoic acid (C22:6 omega-3), and linoleic acid (C18:2 omega-6) were also observed in Cluster 4 (Table 2 and Figure 1). Eicosapentaenoic acid (C20:5 omega-3) and docosahexaenoic acid (C22:6 omega-3), representative omega-3 PUFAs, have been observed to reduce hepatic lipidosis, improve markers of liver damage, and increase insulin sensitivity [31,32]. They appear to exert these beneficial effects on the liver by downregulating pathways related to adipogenesis, inflammation, and fibrogenesis. They are readily incorporated into phospholipid species to maintain cell membrane fluidity and permeability [12]. Thus, a low mass% of omega-3 PUFAs was associated with susceptibility to hepatic lipotoxicity and low insulin sensitivity in Cluster 4, suggesting its association with MAFLD prevalence. Linoleic acid (C18:2 omega-6) is the most abundant PUFA in the ω-6 series, and it is unsaturated by FADS2 in the first step of the conversion process to arachidonic acid (C20:4 omega-6) [33]. FADS2 is a key enzyme in synthesizing arachidonic acid (C20:4 omega-6) from linoleic acid (C18:2 omega-6), and the activity of FADS2 in the plasma has been found to be higher in patients with NAFLD than in healthy participants [34]. In addition, plasma FADS2 activity was found to be positively correlated to BMI, insulin, and visceral fat mass, all of which are closely related to the development and progression of NAFLD [34]. Thus, high BMI and insulin resistance may have increased FADS2 activity in Cluster 4, reducing the mass% of linoleic acid (C18:2 omega-6). The information above suggests that the serum fatty acid composition of Cluster 4, which is strongly associated with MAFLD prevalence, includes a combination of high SFAs, low PUFAs, and increased SCD1 and FADS2 activities; thus, serum fatty acid composition balance may be important in determining MAFLD risk.

The trend test results showed that MAFLD prevalence was in the following order: Cluster 4 > Cluster 3 > Cluster 2 > Cluster 1 (Table 3). In contrast to the association between Cluster 4 and MAFLD prevalence, Clusters 2 and 3 were not associated with MAFLD prevalence in the multivariable analysis (Table 4). The mass% of oleic acid (C18:1 omega-9) in Cluster 2 was higher than that in Cluster 1 (Table 2 and Figure 1). Oleic acid (C18:1 omega-9) is synthesized by unsaturation of stearic acid (C18:0) and through diet and lipolysis. As mentioned above, an increase in SFAs increases SCD1 activity to avoid SFA-induced hepatotoxicity, leading to increased MUFAs [12]. Thus, to avoid hepatotoxicity of stearic acid (C18:0), serum oleic acid (C18:1 omega-9) may have increased in Cluster 2. However, the increase was less than that in Cluster 4, and it may not have been significant enough to be associated with the prevalence of MAFLD. Compared to Cluster 3, Cluster 1 showed differences in the mass% of SFAs and omega-3 PUFAs (Table 2 and Figure 1). SFAs are hepatotoxic, whereas omega-3 PUFAs are hepatoprotective [8,10,11]; therefore, the beneficial effects of omega-3 PUFAs may compensate for the detrimental effects of SFAs, and Cluster 3 was not associated with MAFLD prevalence.

In subjects with MAFLD, GGT and FIB-4 indexes are different among the four clusters, and the clusters can be arranged in terms of decreasing AST and GGT levels as follows: Cluster 4 > Cluster 3 > Cluster 2 > Cluster 1 (Table S3). Therefore, serum fatty acid composition balance may be associated with the severity of MAFLD and, furthermore, may change as MAFLD progresses or recovers. However, the liver function test values and FIB-4 index of the subjects with MAFLD were relatively low (Table 1), indicating that the severity was relatively low and that most of them had simple steatosis. Therefore, further longitudinal studies in MAFLD subjects with fibrosis are needed to clarify the relationship between serum fatty acid composition balance and MAFLD severity and disease course.

In this study, differences in alcohol intake and smoking habits were observed among the clusters (Table 3). Furthermore, the frequencies of eating fruits and sweets were different among the clusters (Table S2). These results may be useful for reducing alcohol consumption, improving smoking cessation, and dietary modification for changing the pattern of serum fatty acid composition balance. However, we were unable to examine the effects of changes in drinking, smoking, and dietary habits on the serum fatty acid composition balance. Moreover, detailed information on dietary habits (e.g., exact intake of carbohydrates, protein, and fat) could not be obtained. Further investigation is needed through longitudinal analysis incorporating detailed information.

The present study had some limitations. It had a retrospective cross-sectional design, and it did not examine the changes in serum fatty acid composition balance. Therefore, further studies are needed to verify the findings of this study by adopting a prospective design in a larger population. Although liver biopsy, the gold standard for fatty liver diagnosis, is more sensitive than other methods, it could not be performed in this study conducted in health screening participants because liver biopsy is invasive. In this study, we used hepatic ultrasonography scanning to diagnose FLD, because this method has a sensitivity of 64% and a specificity of 97% in detecting fatty liver [23]. Therefore, the current study could identify the presence of FLD in the study subjects. However, further studies using liver biopsy are needed to validate the results of this study.

## 5. Conclusions

We determined the serum fatty acid composition balance using the fuzzy c-means method and showed its association with MAFLD prevalence. These results suggest the importance of serum fatty acid composition balance in MAFLD and may contribute to elucidating the pathogenesis of MAFLD. Furthermore, evaluation of serum fatty acid composition balance by the fuzzy c-means method provides more detailed information (e.g., proximity between fatty acid compositional balances) than simple clustering methods (e.g., the k-means method). This method may be useful for lifestyle improvement and for precision and personalized medicine development for the prevention and treatment of MAFLD in the future.

## Figures and Tables

**Table 1:** Clinical characteristics of the study subjects.

	Non-MAFLD (*n* = 257)	MAFLD (*n* = 136)	*p*
Women (%)	93 (36.2)	36 (26.5)	0.055 ^a^
Age (years)	68.6 ± 8.3	61.7 ± 11.3	<0.001
BMI (kg/m^2^)	22.4 ± 2.6	25.9 ± 2.9	<0.001
WC (cm)	82.3 ± 7.8	90.1 ± 6.6	<0.001
HbA1c (%)	5.6 ± 0.7	6.0 ± 1.1	0.002
FBG (mg/dL)	99 (92–107)	101 (94–114)	0.045 ^c^
HDL-C (mg/dL)	68.0 ± 16.0	56.6 ± 14.5	<0.001
LDL-C (mg/dL)	117.9 ± 24.0	130.4 ± 27.4	<0.001
TGs (mg/dL)	84 (66–110)	136 (101–187)	<0.001 ^c^
AST (IU/L)	23.2 ± 7.1	29.3 ± 15.3	<0.001
ALT (IU/L)	20.2 ± 8.9	34.4 ± 20.8	<0.001
GGT (IU/L)	22 (16–35)	33 (26–60)	<0.001 ^c^
FIB-4 index ^d^	-	1.51 (0.49–4.75)	
Diabetes (%)	64 (24.9)	38 (27.9)	0.546 ^a^
Alcohol intake (g/day)	1.4 (0.0–90.0)	3.2 (0.0–90.0)	0.458
Smoking status			
Never	142 (53.8)	70 (51.5)	0.033 ^a^
Ex	103 (39.0)	45 (33.1)	
Current	19 (7.2)	21 (15.4)	
*PNPLA3* genotype			
C/C	83 (32.3)	25 (18.4)	0.015 ^b^
C/G	132 (51.4)	83 (61.0)	
G/G	42 (16.3)	28 (20.6)	
C/C	83 (32.3)	25 (18.4)	0.04 ^a^
C/G or G/G	174 (67.7)	112 (81.6)	

The data are presented as the mean ± standard deviation, median (range), or several subjects (%). ^a^ Fisher exact test; ^b^ Fisher–Freeman–Halton test; ^c^ Mann–Whitney *U* test; Student’s *t*-test was used otherwise. ^d^ FIB-4 index was calculated only in subjects with MAFLD because it is a fibrosis marker for FLD. MAFLD, metabolic dysfunction-associated fatty liver disease; BMI, body mass index; WC, waist circumstance; FBG, fasting blood glucose; HDL-C, high-density lipoprotein cholesterol; LDL-C, low-density lipoprotein cholesterol; TGs, triglycerides; AST, aspartate aminotransferase; ALT, alanine aminotransferase; GGT, gamma-glutamyl transferase; FIB-4 index, fibrosis-4 index; *PNPLA3*, patatin-like phospholipase domain-containing protein 3; FLD, fatty liver disease.

**Table 2:** Differences in the mass% of serum fatty acids and total concentration of serum fatty acids among the four clusters.

Fatty Acid	Cluster 1 (*n* = 112)	Cluster 2 (*n* = 108)	Cluster 3 (*n* = 92)	Cluster 4 (*n* = 88)	*p* ^a^
Myristic acid (C14:0)	0.58 (0.21–1.07)	0.64 (0.22–1.33)	0.70 (0.05–1.34)	0.84 (0.52–1.51)	0.001
Palmitic acid (C16:0)	24.48 (15.89–28.58)	25.09 (21.26–29.15)	26.58 (22.95–31.58)	27.70 (23.18–33.79)	0.001
Stearic acid (C18:0)	9.03 (5.73–11.28)	8.79 (5.55–11.99)	9.15 (7.54–11.88)	8.67 (5.36–11.27)	0.001
Palmitoleic acid (C16:1 omega-7)	1.49 (0.55–2.96)	1.73 (0.74–3.86)	2.05 (1.07–3.81)	2.66 (1.38–6.50)	<0.001
Oleic acid (C18:1 omega-9)	19.45 (14.21–23.21)	22.28 (18.00–29.25)	21.39 (16.49–24.69)	23.55 (16.50–29.74)	0.001
Linoleic acid (C18:2 omega-6)	32.51 (25.94–38.84)	29.43 (27.66–32.11)	26.52 (23.37–28.55)	23.39 (15.87–26.98)	0.001
Alpha- or gamma-linolenic acid (C18:3)	0.71 (0.01–1.47)	0.79 (0.07–1.78)	0.66 (0.27–1.38)	0.66 (0.19–1.28)	0.002
Arachidonic acid (C20:4 omega-6)	5.25 (2.53–8.55)	5.19 (2.18–8.44)	5.67 (2.57–8.90)	5.17 (2.77–8.56)	0.036 ^b^
Eicosapentaenoic acid (C20:5 omega-3)	2.16 (0.47–7.44)	1.84 (0.22–5.50)	2.60 (0.97–7.61)	2.20 (0.74–9.81)	<0.001
Docosahexaenoic acid (C22:6 omega-3)	3.56 (2.00–6.66)	3.49 (1.74–9.81)	4.22 (2.19–8.75)	3.81 (1.52–9.32)	<0.001
Total fatty acids (μg/mL)	2270.3 (1160.8–3716.8)	2424.5 (1453.8–4240.8)	2391.4 (1130.8–3994.8)	2609.5 (1571.3–6130.3)	<0.001

The data are presented as the median (range). ^a^ Kruskal–Wallis test; ^b^ Significance disappeared after Bonferroni’s correction.

**Table 3:** Differences in clinical characteristics among the four clusters.

	Cluster 1 (*n* = 112)	Cluster 2 (*n* = 108)	Cluster 3 (*n* = 92)	Cluster 4 (*n* = 88)	*p*
Women (%)	45 (40.2)	37 (34.3)	26 (28.3)	22 (25.0)	0.107 ^a^
Age (years)	66.2 ± 8.6	66.0 ± 10.6	66.4 ± 9.7	65.3 ± 11.6	0.906
BMI (kg/m^2^)	22.4 ± 2.8	23.5 ± 3.0	24.1 ± 3.4	24.7 ± 3.1	<0.001
WC (cm)	81.9 ± 7.9	84.8 ± 7.8	86.0 ± 8.6	87.5 ± 7.6	<0.001
HbA1c (%)	5.6 ± 0.7	5.7 ± 0.8	5.8 ± 0.9	6.0 ± 1.0	0.041d
FBG (mg/dL)	97 (78–196)	100 (68–244)	101 (81–206)	101 (81–184)	0.022 ^b,d^
HDL-C (mg/dL)	70.3 ± 16.7	63.0 ± 15.3	62.5 ± 15.8	58.6 ± 15.0	<0.001
LDL-C (mg/dL)	125.9 ± 23.2	123.8 ± 23.8	118.6 ± 28.2	120.0 ± 28.1	0.158
TGs (mg/dL)	71.5 (30–270)	103 (48–431)	97 (35–230)	137 (42–508)	<0.001 ^b^
AST (IU/L)	23.1 ± 6.3	24.1 ± 8.7	25.4 ± 10.5	29.3 ± 16.4	<0.001
ALT (IU/L)	21.5 ± 10.6	23.8 ± 17.2	26.0 ± 15.7	30.2 ± 17.5	<0.001
GGT (IU/L)	21 (8–302)	27 (6–185)	27.5 (12–442)	35.5 (15–657)	<0.001 ^b^
MAFLD (%)	22 (19.6)	34 (31.5)	32 (34.8)	48 (54.5)	<0.001 ^a^ <0.001 ^c^
Diabetes (%)	22 (19.6)	27 (25.0)	21 (22.8)	32 (36.4)	0.057 ^a^
Alcohol intake (g/day)	0.0 (0.0–30.0)	0.7 (0.0–60.0)	5.7 (0.0–60.0)	10.7 (0.0–90.0)	<0.001 ^b^
Smoking status					
Never	71 (63.4)	60 (55.6)	44 (47.8)	37 (37.5)	0.025 ^a,d^
Ex	34 (30.4)	40 (37.0)	39 (42.4)	35 (39.8)	
Current	7 (6.3)	8 (7.4)	9 (9.8)	16 (18.2)	
*PNPLA3* genotype					
C/C	34 (30.4)	30 (27.8)	23 (25.0)	22 (25.0)	0.037 ^a,d^
C/G	55 (49.1)	55 (50.9)	50 (54.3)	60 (68.2)	
G/G	23 (20.5)	23 (21.3)	19 (20.7)	6 (6.8)	
C/C	34 (30.4)	30 (27.8)	23 (25.0)	22 (25.0)	0.802
C/G or G/G	78 (69.6)	78 (72.2)	69 (75.0)	66 (75.0)	

The data are presented as the mean ± standard deviation, median (range), or the number of subjects (%). ^a^ Fisher–Freeman–Halton test; ^b^ Kruskal–Wallis test; ^c^ Trend test; one-way ANOVA was used otherwise. ^d^ Significance disappeared after Bonferroni’s correction. BMI, body mass index; WC, waist circumstance; FBG, fasting blood glucose; HDL-C, high-density lipoprotein cholesterol; LDL-C, low-density lipoprotein cholesterol; TGs, triglycerides; AST, aspartate aminotransferase; ALT, alanine aminotransferase; GGT, gamma-glutamyl transferase; MAFLD, metabolic dysfunction-associated fatty liver disease; *PNPLA3*, patatin-like phospholipase domain-containing protein 3.

**Table 4:** Associations of the clusters based on the serum fatty acid composition with the prevalence of MAFLD, according to multivariable logistic regression analysis.

	OR (95% CI) ^a^	*p*
Cluster 1	1	
Cluster 2	1.39 (0.70–2.77)	0.345
Cluster 3	1.56 (0.70–3.44)	0.274
Cluster 4	5.14 (2.03–13.04)	<0.001

^a^ Adjusted for age, sex, BMI, total fatty acid concentration, and *PNPLA3* genotype. MAFLD, metabolic dysfunction-associated fatty liver disease; OR, odds ratio; CI, confidence interval; BMI, body mass index; *PNPLA3*, patatin-like phospholipase domain-containing protein 3.

## Data Availability

The datasets generated and/or analyzed during the current study are not publicly available due to individual privacy but are available from the corresponding authors on reasonable request.

## Supplementary Materials

The following supporting information can be downloaded at: https://www.mdpi.com/article/10.3390/nu15040809/s1, Table S1: Principal component scores of serum fatty acids. Table S2: Differences in dietary habits among the four clusters. Table S3: Differences in liver function test values and FIB-4 index among the four clusters in subjects with MAFLD.
