# Supplementary material for: Serum Fatty Acid Composition Balance by Fuzzy C-Means Method in Individuals with or without Metabolic Dysfunction-Associated Fatty Liver Disease
Source: Nutrients. 2023 Feb 4;15(4):809. doi: 10.3390/nu15040809 (PMC9960614; doi:10.3390/nu15040809)
Supplement: Supplementary file 1 [file nutrients-15-00809-s001.zip › nutrients-2182829-supplementary.pdf]

Supplementary Information

Table S1. Principal component scores of serum fatty acids.

Fatty acids	First principal component	Second principal component
Myristic acid (C14:0)	0.492	−0.082
Palmitic acid (C16:0)	0.659	0.000
Stearic acid (C18:0)	−0.057	0.334
Palmitoleic acid (C16:1 omega-7)	0.682	−0.014
Oleic acid (C18:1 omega-9)	0.627	−0.776
Linoleic acid (C18:2 omega-6)	−0.963	−0.188
Alpha- or gamma-linolenic acid (C18:3)	−0.089	−0.193
Arachidonic acid (C20:4 omega-6)	−0.027	0.448
Eicosapentaenoic acid (C20:5 omega-3)	0.090	0.793
Docosahexaenoic acid (C22:6 omega-3)	0.104	0.628
% Variance	24.80	20.17
Cumulative variance	24.80	44.97

Table S2. Differences in dietary habits among the four clusters.

Frequency of consumption of the foods		*N*	Cluster 1	Cluster 2	Cluster 3	Cluster 4	*P*
Rice, bread, and/or noodles (%)	≥600 g/day	199	34 (54.8)	33 (55.9)	31 (70.5)	20 (58.8)	0.382
Meat dishes (%)	≥1 time/day	199	41 (66.1)	42 (71.2)	31 (70.5)	25 (73.5)	0.888
Fish dishes (%)	≥1 time/day	400	70 (62.5)	64 (59.3)	55 (59.8)	65 (73.9)	0.131
Egg dishes (%)	≥1 time/day	199	33 (53.2)	43 (72.9)	30 (68.2)	21 (61.8)	0.140
Vegetables (%)	≥2 times/day	400	79 (70.5)	65 (60.2)	52 (56.5)	55 (62.5)	0.188
Fruits (%)	≥1 time/day	199	46 (74.2)	26 (44.1)	24 (54.5)	24 (70.6)	0.003
Sweets (%)	≥2 times/week	400	85 (75.9)	77 (71.3)	66 (71.7)	46 (52.3)	0.003
Salted foods (%)	≥1 time/day	400	48 (42.9)	43 (39.8)	40 (43.5)	42 (47.7)	0.741

Fisher–Freeman–Halton test.

Table S3. Differences in liver function test values and FIB-4 index among the four clusters in subjects with MAFLD.

	Cluster 1 (*n* = 22)	Cluster 2 (*n* = 34)	Cluster 3 (*n* = 32)	Cluster 4 (*n* = 48)	*P*
AST (IU/L)	24.1 ± 4.4	27.3 ± 13.3	29.9 ± 15.2	32.8 ± 19.0	0.126<br>0.002[b]
ALT (IU/L)	27.7 ± 12.6	33.8 ± 27.2	36.8 ± 20.8	36.2 ± 18.3	0.382<br>0.055[b]
GGT (IU/L)	28.5 (8–70)	34 (15–185)	33.5 (14–442)	38 (16–657)	0.024[a]<br>0.012[b]
FIB-4 index	1.59 (0.55–3.76)	1.26 (0.59–2.56)	1.41 (0.49–4.31)	1.62 (0.54–4.75)	0.019[a]<br>0.130[b]

The data are presented as the mean ± standard deviation, median (range), or the number of subjects (%).
[a]Kruskal–Wallis test; [b]Trend test; one-way ANOVA was used otherwise.
MAFLD, metabolic dysfunction-associated fatty liver disease; AST, aspartate aminotransferase; ALT, alanine aminotransferase; GGT, gamma-glutamyl transferase; FIB-4 index, fibrosis 4 index.
